# Food hygiene practice and associated factors among street food vendors in Addis Ababa; Ethiopia; mixed approach study

**DOI:** 10.3389/ijph.2026.1609008

**Published:** 2026-05-14

**Authors:** Abduselam Ahmed Abdela, Tufa Kolola Huluka, Mecha Aboma Yebassa, Samuel Dessu Sifer

**Affiliations:** 1 Department of Public Health, Yanet College, Addis Ababa, Ethiopia; 2 Departments of Public Health, College of Medicine and Health Sciences, Ambo University, Ambo, Ethiopia; 3 School of Public Health, Yekatit 12 Hospital Medical College, Addis Ababa, Ethiopia

**Keywords:** Addis Ababa, Ethiopia, food hygiene, street food vendor, vending site

## Abstract

**Objectives:**

This study aimed to assess food hygiene practices and associated factors among street food vendors in Addis Ababa.

**Methods:**

A community-based cross-sectional study with mixed methods was conducted from 1–30 March 2024. A total of 337 vendors participated in the quantitative survey, and 25 took part in qualitative interviews and focus group discussions. Consecutive sampling was applied for the quantitative component and convenience sampling for the qualitative part. Data were collected using structured questionnaires with observational checklists and unstructured guides for qualitative interviews. Multivariable logistic regression identified predictors at p < 0.05, and qualitative data were analyzed thematically.

**Results:**

Good hygiene practice was observed in 33.2% (95% CI:28.2–38.3). Significant factors included training (AOR: 2.43), knowledge (AOR:3.44), uninterrupted water supply (AOR: 2.52), professional visits (AOR: 5.42), and work experience >1 year (AOR: 4.68). Qualitative findings revealed barriers including inadequate water and sanitation, poor working conditions, lack of training, financial constraints, and customer pressures.

**Conclusion:**

Hygiene practices were generally low. Findings emphasize the need for targeted interventions in training, supervision, and infrastructure to improve food hygiene among street food vendors.

## Introduction

Street food vending plays a critical role in food security and urban livelihoods in developing countries by providing affordable meals and employment opportunities for economically disadvantaged populations [1]. In addition to meeting daily dietary needs, the sector contributes to local economies and serves as an important source of income, particularly for women with limited access to capital and formal employment [2–4]. Rapid urbanization, unemployment, and poverty have further increased reliance on street foods, driven by their affordability, accessibility, and distinctive flavors [5, 6].

Despite these socioeconomic benefits, street food vending is frequently associated with unsafe food handling practices that expose consumers to foodborne diseases [7, 8]. Food safety remains a major global public health concern, particularly in low- and middle-income countries [9, 10]. The Foodborne Epidemiology Reference Group (FERG) estimates that unsafe food causes approximately 600 million illnesses and 420,000 deaths annually, with diarrheal diseases accounting for the largest share of this burden [11–14]. A substantial proportion of foodborne disease outbreaks in developing countries—estimated at 10–20%—has been attributed to contaminated street foods [15–17], highlighting the public health relevance of this sector in rapidly urbanizing settings.

Consistent with these global patterns, evidence from Africa and Asia indicates widespread poor hygiene practices among street food vendors. Studies from Ethiopia and Ghana report that only 2.8% and 15% of vendors, respectively, wash their hands before food handling [18–20], while observations from Gondar, Ethiopia show that approximately half of vendors do not wash hands with soap during food preparation or service [21]. Inadequate protection of food from flies and environmental contamination is also common, with 94% of Ethiopian vendors and 42.1% of vendors in Zanzibar failing to properly cover food [22–24]. Structural challenges further exacerbate these risks, as over half of Ethiopian street food stalls operate near open drains [25], and limited access to clean water and hand washing facilities has been reported in China and Kenya [26–28].

National-level reviews in Ethiopia further identify limited vendor knowledge, inadequate infrastructure, and weak regulatory enforcement as persistent constraints to safe street food practices [29–32]. Together, these findings suggest that street food hygiene is shaped by a complex interaction of individual behaviors, environmental conditions, and institutional factors. However, existing studies have largely focused on measuring the prevalence of hygiene practices, with limited exploration of vendors’ perceptions, decision-making processes, and the contextual and regulatory environments that influence behavior [14, 16, 18, 19].

Street food vending plays an important role in providing affordable and accessible meals in many low- and middle-income countries. In Addis Ababa, the practice is widespread, serving a large proportion of urban residents—particularly low-income populations and daily laborers—while also supporting livelihoods through informal employment. Despite its socioeconomic importance, street food vending in the city often operates under informal conditions, with limited infrastructure, inadequate access to water and sanitation, and inconsistent adherence to food safety standards. Although Ethiopia has national food safety and public health regulations, enforcement among street food vendors remains challenging due to resource constraints and the informal nature of the sector. Limited routine inspection and training increase the risk of unsafe food handling practices, raising public health concerns and underscoring the need for context-specific evidence on food hygiene practices and their determinants in Addis Ababa.

Despite a growing body of research on street food safety in Ethiopia, evidence remains fragmented and predominantly quantitative, with minimal integration of qualitative insights. Notably, no prior study has employed a mixed-methods approach to comprehensively assess determinants of food hygiene practices among street food vendors in Addis Ababa, where rapid urban growth and increasing reliance on ready-to-eat foods have expanded the sector substantially. This gap limits understanding of why unsafe practices persist despite existing regulations and knowledge of food safety risks. Therefore, this study aims to assess food hygiene practices among street food vendors in Addis Ababa and identify factors associated with safe and unsafe practices using a mixed-methods approach. By integrating quantitative and qualitative data, the study seeks to generate context-specific evidence to inform targeted public health interventions and policy responses.

## Methods

### Study design, area and period

A community-based cross-sectional study with a mixed-methods approach was conducted in Addis Ababa from 1 March to 30 March 2024. Addis Ababa is divided into 11 sub-cities and had an estimated population of 5,704,000 in 2024.

### Population

All street food vendors operating in Addis Ababa constituted the source population. Street food vendors working within the selected sub-cities formed the study population. Vendors engaged in the preparation and/or sale of ready-to-eat street foods were eligible for inclusion, whereas mobile vendors and those selling exclusively pre-packaged foods were excluded from the study.

### Sample size determination and procedure

The sample size was calculated using the single population proportion formula:

$$n=\frac{{\left(z_{\alpha /2}\right)}^{2}*p\left(1-p\right)}{d^{2}}$$

where *n* is the required sample size, *Z*α/2 is the standard normal value corresponding to a 95% confidence level (1.96), *p* is the estimated prevalence of good food hygiene practice (27.5%) obtained from a previous study [27], and *d* is the margin of error (0.05). After calculating the initial sample size, a 10% non-response rate was added. Accordingly, the final sample size for the study was 337 participants.

For the qualitative component, participant numbers for in-depth interviews were determined using the principle of saturation, where no new themes or insights emerge. The researcher iteratively reviewed transcripts, identifying and verifying categories as data collection progressed. Earlier transcripts were revisited to ensure that additional interviews were not yielding novel information. Once saturation was achieved, a total of 18 in-depth interviews and three focus group discussions were conducted, providing diverse perspectives relevant to the study objectives. This approach ensured comprehensive coverage of participants’ experiences while capturing the full range of themes and insights.

Of the 11 sub-cities in Addis Ababa, four (Addis Ketema, Kolfe Keraniyo, Kirkos, and Lemi Kura) were selected using simple random sampling. Within each selected sub-city, three vending sites were chosen through convenience sampling. Sample sizes were proportionally allocated based on preliminary estimates of the number of vendors at each site, and participants were recruited consecutively to ensure representation across all selected locations.

For the qualitative component, participants were selected using purposive sampling to capture a range of perspectives relevant to the study objectives. Selection criteria included age, sex, type of street food activity, and level of involvement in food preparation and sale. While the use of purposive and consecutive sampling may limit statistical representativeness, this approach was appropriate for in-depth exploration of contextual factors influencing hygiene practices. To mitigate potential selection bias and enhance diversity of perspectives, vendors were recruited from multiple districts across Addis Ababa, and interviews were conducted on different days of the week and at varying times of day to capture variation in operating conditions, customer flow, and hygiene practices.

### Operational definitions

Food hygiene knowledge: Vendors scoring ≥8 out of 10 knowledge questions were considered to have good knowledge, while those scoring <8 were classified as having poor knowledge [15].

Hygiene practice level: Vendors scoring ≥17 out of 21 hygiene practice questions were considered to have good practices, while those scoring <17 were classified as having poor hygienic practices [15].

### Data collection tool and procedure

Quantitative data were collected using a pretested, structured, interviewer-administered questionnaire, and an observational checklist was employed by trained data collectors. Observations were conducted prior to administering the questionnaires to ensure that the data collectors could record actual food handling practices without influencing participant responses. The data collection tool was prepared in English, translated into Amharic for clarity, and back-translated into English by two language experts to ensure consistency. The instrument was adapted from previous studies [14, 17].

For the qualitative component, baseline demographic and contextual information was collected before data collection. In-depth interviews (IDIs) and focus group discussions (FGDs) were conducted to obtain rich insights. IDIs were conducted face-to-face in private settings to ensure confidentiality and typically lasted 30–45 min, while FGDs, each comprising eight participants and lasting 90–120 min, were held in neutral venues with informed consent and audio recording. A trained facilitator led the sessions using a semi-structured guide, and a note-taker documented key points, non-verbal cues, and group dynamics. Field notes captured contextual details and researcher reflections. The guides explored participants’ experiences, perceptions, and challenges, with flexibility to probe emerging issues while addressing study objectives.

### Integration of quantitative and qualitative data

Quantitative and qualitative data were collected concurrently and analyzed separately. Integration was conducted at the interpretation stage using a side-by-side comparison approach. Quantitative findings on hygiene practices and associated factors were compared with qualitative themes to identify convergence and divergence. Qualitative data were used to explain statistically significant associations, contextualize non-significant findings, and clarify underlying mechanisms. Cross-validation was achieved by examining consistency between self-reported quantitative responses and qualitative accounts of routine practices and constraints, thereby strengthening the credibility and interpretability of the findings.

### Data processing and analysis

Quantitative data were entered into Epi Info version 7 and exported to SPSS version 25 for analysis. Food hygiene knowledge was assessed using ten closed-ended questions, with scores based on correct responses. Food handling practices were evaluated using 21 items, with each standard practice scored as 1 and unsafe practices as 0. The internal consistency of the instrument was assessed using Cronbach’s alpha, yielding a value of 0.78, indicating acceptable reliability. Bivariate and multivariable logistic regression analyses identified associations between independent and dependent variables. Variables with p < 0.25 in bivariate analysis were entered into the multivariable model. Crude odds ratios (ORs), adjusted odds ratios (AORs), and 95% confidence intervals (CIs) assessed the strength and significance of associations. Variables with p < 0.05 in the multivariable model were considered statistically significant.

Qualitative data were transcribed verbatim, translated into English, and imported into Atlas ti for analysis. Thematic analysis began with repeated transcript readings to ensure familiarity. Codes were generated inductively and deductively, grouped into categories, and refined into overarching themes. Themes were interpreted in relation to study objectives and illustrated with participant quotes to enhance credibility. Trustworthiness was maintained through systematic analysis and careful interpretation.

### Trustworthiness of qualitative data

To ensure credibility, qualitative data were analyzed systematically using thematic coding. Preliminary findings and emergent themes were shared with a subset of vendors (member checking) to confirm accurate representation of their experiences. Triangulation was conducted by comparing qualitative insights with quantitative results, enhancing interpretive validity. Additionally, coding and theme development were independently reviewed by a second researcher (peer debriefing) to minimize bias and strengthen the reliability and trustworthiness of the analysis.

### Ethical considerations

Ethical clearance was obtained from the Institutional Review Board of Yanet College (Ref: YC/IRB/214/2024). Verbal consent was obtained from participants, and privacy, confidentiality, and anonymity were ensured. The study was conducted in accordance with the Declaration of Helsinki.

## Results

### Socio demographics characteristics of street food vendors

A total of 337 street food vendors participated (response rate: 100%), with the majority (97.6%) being female. The mean age was 31.5 ± 6.3 years, and 78% were married. Over one-third had completed primary education, and 24.6% had less than 1 year of vending experience (Table 1).

**TABLE 1:** Socio-demographic characteristics of street food vendors in Addis Ababa (Ethiopia, 2024).

Variables	Category	Frequency (n)	Percentage (%)
Sex	Male	8	2.4
	Female	329	97.6
Age	18–30	171	50.74
	31–40	130	38.57
	41–50	36	10.68
Marital status	Single	51	15.1
	Married	263	78
	Divorced	23	6.82
Educational level	No formal education	45	13.4
	Primary education	136	40.3
	Secondary	68	20.2
	Certificate	32	9.5
	Diploma	33	9.8
	Degree	23	6.8
Monthly income	<2,500 Ethiopian Birr	116	34.42
	2,501–3,500 Ethiopian Birr	159	47.18
	>3,501 Ethiopian Birr	62	18.39
Work experience	Less than 1 year	83	24.6
	1–3 years	101	30
	More than 3 years	153	45.4

### Water supply used by the street food vendors

Among 337 participants, most vendors (94.1%) obtained water from kiosks, while 5.9% relied on water from home. Nearly all (98.8%) paid for water services, and for storage, 75% used jerrycans and 25% used buckets. Over two-thirds (67.9%) reported experiencing interrupted water supply, which affected their ability to maintain hygiene practices.

These quantitative findings were reinforced by qualitative insights. Vendors described how limited water availability constrained hand washing and utensil cleaning: one 29-year-old participant explained, “I bring water from home every morning, but if it finishes before noon, I just have to manage without it.” Similarly, a 41-year-old vendor noted, “Sometimes we buy bottled water to wash hands, but it’s too expensive to do that every day.” Lack of nearby hand washing facilities also emerged as a barrier, with a 36-year-old woman stating, “There is no hand washing station nearby, and walking far just to wash hands is not practical when we have customers waiting.” The practical impact on utensil hygiene was highlighted by a 26-year-old participant: “We only wash utensils once or twice a day because water is limited.”

### Training on food preparation skill

Qualitative insights provide context for these patterns. A 38-year-old vendor explained, “No one has ever told us how to prepare or store food safely. We do it the way we learned from others,” illustrating the reliance on informal learning. Similarly, another participant noted, “They say you need a license and training, but I’ve never been given a chance or support,” highlighting gaps in access to formal training. Limited knowledge about basic hygiene concepts was also evident: a 28-year-old vendor reported, “I’ve never heard of cross-contamination [menekakat]. We cut meat and vegetables on the same board because we only have one.” Together, these quantitative and qualitative findings demonstrate that lack of formal training and reliance on informal learning contributes to unsafe hygiene practices among street food vendors.

### Hygienic practice of vendors during preparation and processing of foods

Food preparation and handling practices varied among vendors. Nearly half (45.7%) prepared food both at home and at the vending site, while 42.4% prepared food exclusively at the stall and 11.9% at home. Most of them (96.1%) cooked food in the morning prior to sales, with only 3.9% preparing food during selling hours. Regarding food handling, 65.3% of vendors handled ready-to-eat foods using bare hands, whereas 34.7% used utensils. In terms of food storage, 50.7% kept food items openly at the stall, while 49.3% used covered containers. Leftover food was discarded by 65.3% of vendors, consumed at home by 24.9%, and resold the following day by 9.8%. For cross-contamination prevention, 75.7% reported using separate utensils for raw and cooked foods, while 24.3% did not. Utensil sanitization practices showed that 75.4% cleaned utensils once daily, 18.7% twice daily, and 5.9% three times per day.

### Personal hygiene practice of street food vendor

Most vendors (95%) reported washing hands before handling food, but only 123 (36.5%) washed hands after handling raw food. All 337 (100%) washed hands after using the toilet, yet only 66 (19.5%) used soap. Observations showed that just 27 vendors (8%) frequently washed hands. Only 94 (27.9%) wore aprons, and 79.5% did not cover their hair. Long nails were observed in 212 (63.2%), and 221 (65.6%) wore hand jewelry while cooking. About 328 (97.3%) handled money without washing hands, 235 (69.7%) used bare hands for cooked food, and 10% had open skin lesions. After data collection, health education sessions were conducted to address critical hygiene gaps.

### Environmental sanitation status of vending site

Only 106 vendors (31.5%) worked in a clean environment, and 95% operated in areas without designated refuse sites. Waste bins were available at 38.3% of vending sites, and 64.7% of vendors disposed of waste via open dumping. Handwashing facilities were present at 197 sites (58.7%), but soap was available at only 139 units (41.2%). Overall, just 68 vendors (20.2%) operated under good environmental sanitation conditions.

Qualitative insights help explain these patterns. A 34-year-old vendor described, “The area is very dusty, and vehicles pass by all the time. I try to cover my food, but it still gets dirty,” illustrating the challenge of maintaining cleanliness in busy, exposed locations. Similarly, a 28-year-old participant noted, “I work under a tree near the market. There’s no clean place to cut vegetables or keep cooked food,” highlighting the lack of appropriate food preparation surfaces. Adverse conditions were echoed by a 42-year-old woman: “When it rains, the place gets muddy and dirty. But we can’t stop working because of that.” Lack of nearby sanitation facilities further constrained hygiene practices, as a 33-year-old male vendor reported, “There are no toilets nearby. If we need to go, we either wait or use unsafe places.” Together, these quantitative and qualitative findings demonstrate that limited infrastructure, poor waste management, and inadequate sanitation facilities significantly hinder vendors’ ability to maintain safe environmental conditions, even when they are aware of proper hygiene practices.

### Food handling knowledge of street food vendors

Only one-fourth (25%) of street food vendors demonstrated good knowledge of food handling practices. While all vendors (100%) recognized the importance of hand hygiene, 77.7% were unaware of the benefits of separating raw and cooked foods. Additionally, 81.3% lacked knowledge about the risks of uncovered hand wounds, and 81% did not know that individuals with infections should refrain from food handling (Table 2).

**TABLE 2:** Food handling Knowledge Assessment for Street Food Vendors at Addis Ababa (Ethiopia, 2024).

Assessment domain	Categories	Frequency (n)	Percentage (%)
Hand hygiene can prevent food contamination	Yes	337	100
Contact between raw and cooked food can contribute for food contamination	Yes	75	22.3
Proper cleaning and sanitation of utensil can prevent food contamination	Yes	296	87.8
Is food handlers with disease like diarrhea, sore throat should avoided from handling food	Yes	63	18.7
Cooked food could have disease causing microorganism if not kept well	Yes	174	51.6
Spoiled food have change in color and testes	Yes	245	72.7
Can open wound be a source of food contamination	Yes	63	18.7
Dish drying cloth can be risk for contamination if not kept hygienically	Yes	66	19.5
Reheating food can kill disease causing micro-organism	Yes	337	100
Covering food can prevent contamination	Yes	280	83.1
Over all food hygiene knowledge score	Good	85	25.2
	Poor	252	74.8

Qualitative insights help explain these gaps. A 25-year-old vendor stated, “I don’t know why we should keep raw and cooked food separate. I just put them in one place to save space,” reflecting the high proportion unaware of cross-contamination risks. Similarly, a 28-year-old participant reported, “I wash my hands only when they look dirty. I didn’t know you have to wash before touching food every time,” illustrating partial understanding of hand hygiene. Limited awareness of broader food safety risks was captured by a 40-year-old woman: “I thought food poisoning only happens when food smells bad [metfo shita]. If it smells okay, I sell it.” Knowledge gaps regarding proper food storage were also evident, with a 41-year-old vendor noting, “We don’t know the right temperature to store food. We just cover it with cloth to keep it warm.”

These qualitative narratives contextualize the quantitative findings, showing that limited food handling knowledge underlies unsafe practices, including improper storage, insufficient hand hygiene, and failure to separate raw and cooked foods.

### Enforcement of food safety regulations among street food vendors

Enforcement of food safety regulations was limited among street food vendors. Over half of the vendors (202, 59.9%) were unregistered, and 189 (56.1%) had not been visited by health professionals. Additionally, the majority (314, 93.2%) had not undergone medical checkups in the past 6 months. These quantitative findings were reflected in vendors’ accounts. A 37-year-old woman explained, “No one has ever asked me why I don’t wear gloves or apron, so I think it’s not necessary,” illustrating how limited regulatory oversight and lack of health professional visits reduce adherence to recommended hygiene practices. Together, the findings suggest that insufficient enforcement, irregular HP visits, and absent medical checkups contribute to gaps in hygiene compliance, despite vendors’ awareness of basic food safety principles.

### Economic constraints affecting hygiene compliance

Economic constraints were a major barrier to proper food hygiene among street food vendors. Many vendors reported that daily expenses for gloves, soap, and food covers were unaffordable. A 26-year-old participant explained, “Buying gloves, soap, or covers every day is not possible with the little we earn.” Similarly, one vendor noted, “Sometimes I reuse cooking oil for 3 or 4 days because I can’t afford new oil every time.” Concerns about profit loss were also common: a 45-year-old woman stated, “Food is expensive, so if I throw it away, I lose profit. I sell leftovers the next day.” Limited financial support further constrained their ability to maintain safe practices, as a 40-year-old participant added, “We would like to improve, but no one supports us with loans or materials to start properly.

### Customer and market pressures on hygiene practices

Customer expectations and market pressures influenced vendors’ hygiene practices. Many vendors reported prioritizing speed and affordability over strict hygiene compliance. A 32-year-old participant explained, “Customers don’t want to wait long, so I serve fast even if I haven’t washed my hands properly.” Another vendor noted, “People buy from me because the food is cheap, not because it is clean.” Focus group discussions further emphasized the trade-off between cleanliness and business: “When we clean too much or raise prices, customers leave and go to cheaper places.”

### Food hygienic practices among street food vendors

The prevalence of good hygienic practices among street food vendors in Addis Ababa was 33.2% (95% CI: 28.2–38.3). While 123 vendors (36.5%) reported washing their hands after handling raw foods, only 27 (8%) were observed washing hands frequently during cooking and serving. This discrepancy between self-reported and observed behaviors suggests potential social desirability and reporting biases, where vendors may overestimate compliance when responding to surveys. Observational data provide a more objective assessment of routine practices, revealing that actual hygiene behaviors are substantially lower than reported. These findings highlight the importance of triangulating self-report with direct observation in assessing food handling practices, as reliance on reported behaviors alone may overstate adherence to hygiene standards. Additionally, 76.9% stored cooked food in uncovered utensils, 81.9% cleaned utensils in visibly dirty water, and 56.4% exposed food to flies (Table 3).

**TABLE 3:** Street Food vendors’ Hygienic practice in Addis Ababa, (Ethiopia, 2024).

Variables	Categories	Frequency (n)	Percentage (%)
Hand washing before handling foods	Yes	320	95
Hand washing after handling raw foods	Yes	123	36.5
Hand washing after visiting toilet	Yes	337	100
Prepare food when you feel ill	Yes	337	100
Type of water used to wash hands	Soap running water	40	11.9
	Running water only	28	8.3
	Water in container	243	72.1
	Water in container and soap	26	7.7
Hand washing after sneezing or coughing	No	337	100
How did they sell food	From tray with covering	55	83.7
	From tray with no covering	282	16.3
Food stored in covered container	Yes	78	23.1
Vendor blow air into plastic bag used to pack food	Yes	76	22.6
Vendor frequently wash hands	Yes	27	8
Cleaning water visibly dirty	Yes	276	81.9
Proper disposal of waste	​	112	33.2
Store cooked food at safe temperature	Yes	102	30.3
Keep food off the floor	Yes	141	41.8
Avoid cross-contamination between utensils	Yes	119	35.3
Maintain personal hygiene (hair tied, nails short)	Yes	116	34.4
Keep vending area free of animals/insects	Yes	112	33.2
Clean and sanitize chopping boards regularly	Yes	102	30.3
Dispose of leftover food safely	Yes	141	41.8
Clean food preparation tables before use	Yes	125	37.1
Cover prepared food during transport	Yes	132	39.2

### Factors associated with hygienic practice of street food vendors

Multivariable logistic regression identified several factors significantly associated with good food hygiene practices among street food vendors, even after adjusting for confounders. Vendors with 1–3 years of experience had 4.68 times higher odds of good hygiene compared to those with less than 1 year (AOR: 4.68; 95% CI: 1.96–11.14), and those with over 3 years of experience had 8.87 times higher odds (AOR: 8.87; 95% CI: 3.72–21.15). Continuous water supply increased the likelihood of good hygiene by 2.52 times (AOR: 2.52; 95% CI: 1.37–4.62). Formal food handling training raised the odds by 2.43 times compared to learning through observation (AOR: 2.43; 95% CI: 1.12–5.27). Regular visits from health professionals increased the odds by 5.42 times (AOR: 5.42; 95% CI: 2.90–10.12), and vendors with good food hygiene knowledge were 3.44 times more likely to demonstrate proper practices than those with poor knowledge (AOR: 3.44; 95% CI: 1.60–7.38) (Table 4).

**TABLE 4:** Factors associated with hygienic practice of street food vendors in Addis Ababa (Ethiopia, 2024).

Variables	Category	Status of hygienic practice		COR (95%CI)	AOR (95%CI)
		Good	Poor		
Work experience	Less than 1 year	12	71	1	1
	1–3 years	37	64	3.42 (1.64, 7.12)^a^	4.68 (1.96, 11.14)^b^
	More than 3 years	63	90	4.14 (2.07, 8.26)^a^	8.87 (3.72, 21.15)^b^
Uninterrupted water source	Yes	48	60	2.06 (1.28, 3.32)^a^	2.52 (1.37, 4.62)^b^
	No	64	165	1	1
Ways of acquiring food preparation skill	Formal training	50	41	3.37 (1.90,5.95)^a^	2.43 (1.12, 5.27)^b^
	Parents	28	90	0.86 (0.48,1.53)^a^	0.87 (0.43, 1.79)
	Observation	34	94	1	1
Visited by HP	No	28	161	1	1
	Yes	84	64	7.54 (4.50, 12.65)^a^	5.42 (2.90, 10.12)^b^
Registered to sell food	No	49	153	1	1
	Yes	63	72	2.73 (1.71, 4.35)^a^	0.88 (0.39, 2.00)
Knowledge on food handling	Poor	68	184	1	1
	Good	44	41	2.90 (1.74, 4.82)^a^	3.44 (1.60, 7.38)^b^
Environmental sanitation status	Poor	77	192	1	1
	Good	35	33	2.64 (1.53, 4.55)^a^	1.53 (0.69, 3.38)

^a^ Indicates variables having P-value less than 0.25 in bivariate analysis.

^b^ Indicates variables having P-value less than 0.05 in multivariable analysis.

### Integration of quantitative and qualitative findings on determinants of street food hygiene practices

Continuous water supply was significantly associated with good hygiene practices, increasing the likelihood by 2.52 times. Qualitative findings provide context to this association, revealing that vendors face practical challenges in maintaining adequate water throughout the day. Many rely on water brought from home or purchased bottled water, which is often insufficient or costly, limiting both hand washing and utensil cleaning. The lack of nearby hand washing facilities further constrains hygiene practices, as walking long distances to access water is impractical during busy service hours. Together, these insights explain how limited water availability and infrastructural constraints directly impact vendors’ ability to maintain proper hygiene, complementing the quantitative results.

Qualitative interviews and focus group discussions underlined the practical challenges behind these numbers. One 29-year-old vendor explained, “I bring water from home every morning, but if it finishes before noon, I just have to manage without it.” Similarly, a 41-year-old vendor noted, “Sometimes we buy bottled water to wash hands, but it’s too expensive to do that every day.” Lack of nearby hand washing facilities further constrained hygiene practices, as a 36-year-old woman from FGD mentioned, “There is no hand washing station nearby, and walking far just to wash hands is not practical when we have customers waiting.” Limited water availability also affected utensil cleaning, with a 26-year-old participant stating, “We only wash utensils once or twice a day because water is limited.”

Regular visits from health professionals were strongly associated with good hygiene practices, increasing the odds by 5.42 times. Qualitative findings provide context for this effect, revealing that vendors often underestimate the importance of hygiene when external monitoring or guidance is absent. Some vendors reported that, without oversight, they do not consistently use protective equipment such as gloves or aprons. Focus group discussions further highlighted the tension between maintaining strict hygiene and sustaining business, as vendors may limit cleaning or protective measures to avoid inconveniencing customers or raising prices. These insights help explain how regular health professional visits can reinforce hygiene practices by providing guidance, accountability, and encouragement, complementing the quantitative results.

Vendors with good food hygiene knowledge were 3.44 times more likely to demonstrate proper practices than those with poor knowledge. Qualitative findings help explain this association, showing that limited understanding of food safety leads to unsafe handling practices. Many vendors were unaware of the need to separate raw and cooked foods, wash hands consistently before handling food, or maintain proper storage conditions. Misconceptions about foodborne illness were also common, with some vendors believing that food is safe as long as it looks or smells acceptable. These knowledge gaps directly affect hygiene practices, reinforcing the quantitative finding that better food hygiene knowledge significantly increases the likelihood of safe food handling.

Formal food handling training was significantly associated with good hygiene practices, increasing the odds by 2.43 times compared to vendors who learned informally through observation. Qualitative findings provide important context for this association. Many vendors reported that they had never received structured guidance on safe food preparation or storage and instead relied on practices learned informally from peers or through experience. Participants also indicated limited access to licensing support and formal training opportunities, suggesting systemic gaps in capacity-building efforts. In addition, misunderstandings about basic food safety principles such as cross-contamination and the importance of using separate equipment for raw and cooked foods were common, often compounded by resource constraints like limited utensils. These qualitative insights help explain the quantitative findings, indicating that formal training not only improves knowledge but also equips vendors with practical skills and risk awareness that informal learning alone may not provide (Table 5).

**TABLE 5:** Integrated Quantitative and Qualitative Findings on determinants of Hygiene Practices among Street Food Vendors in Addis Ababa (Ethiopia, 2024).

Variables	AOR (95%CI)	Theme	Illustrative quotes
Having uninterrupted water source	2.52 (1.37, 4.62)	Limited water access	“I bring water from home every morning, but if it finishes before noon, I just have to manage without it.” (29-year-old woman) vendor noted, “sometimes we buy bottled water to wash hands, but it’s too expensive to do that every day.” (41-year-old woman) “There is no hand washing station nearby, and walking far just to wash hands is not practical when we have customers waiting.” (36-year-old) “We only wash utensils once or twice a day because water is limited” (26-year-old woman)
Having food handling training	2.43 (1.12, 5.27)	Lack of formal training	“No one has ever told us how to prepare or store food safely. We do it the way we learned from others” (38 years old woman) “They say you need a license and training, but I’ve never been given a chance or support.” (28-yearold woman)
Visited by HP	5.42 (2.90, 10.12)	Limited health professional visit	“No one has ever asked me why I don’t wear gloves or apron, so I think it’s not necessary” ( 37-year-old woman
Good knowledge on food handling	3.44 (1.60, 7.38)	Limited food handling knowledge	“I don’t know why we should keep raw and cooked food separate. I just put them in one place to save space.” ( 25 years old woman , “I wash my hands only when they look dirty. I didn’t know you have to wash before touching food every time.” 28 years old woman) “We don’t know the right temperature to store food. We just cover it with cloth to keep it warm.” (41 years old woman)

## Discussion

The prevalence of good hygiene practices among street food vendors in Addis Ababa was 33.2%, comparable to findings from Gedeo Zone, Southern Ethiopia (31.5%) [18], but lower than reports from Dessie (53%), Gondar (49.1%), and Gojam (51.4%) [19–21]. These differences likely reflect structural and regulatory disparities rather than random variation. Settings with higher vendor registration and routine medical checkups appear to create an enabling environment for hygiene compliance, whereas lower registration (40%) and medical checkups (6.8%) in Addis Ababa suggest weaker regulatory oversight. Qualitative findings further illuminate these mechanisms, highlighting infrequent inspections, limited access to hand washing facilities, and pressure to prioritize customer demand over hygiene, which collectively constrain safe food-handling behaviors.

International comparisons highlight the role of structural factors. Hygiene prevalence in Addis Ababa was lower than in Brazil, Myanmar, Bangladesh, Poland, Benin City, Nigeria, and Alexandria, Egypt [10, 22, 26, 32–34], likely reflecting differences in vendor registration, water access, and study inclusion criteria. Conversely, it exceeded rates in Tharaka Nithi County, Kenya (12%) and Shashamene, Ethiopia [27], suggesting that urban market dynamics and informal oversight may support better practices. These findings highlight that food hygiene is influenced more by infrastructure, regulation, and economic context than by individual behavior alone.

Several determinants of hygiene practices align with plausible behavioral and structural mechanisms. Longer work experience was associated with better hygiene practices, consistent with studies from Zanzibar, Kiambu County in Kenya, Bishoftu, and Dessie, Ethiopia [28, 29, 34–36]. Experienced vendors may develop practical strategies to maintain hygiene under resource constraints, whereas newer vendors lack such experiential learning.

Continuous access to water was associated with better hygiene practices, consistent with findings from Gedeo Zone, Southern Ethiopia, and Florianopolis, Brazil [18, 30, 37]. Qualitative insights explain this association: vendors reported rationing water during peak hours, prioritizing cooking over cleaning, and sometimes skipping hand washing due to limited access or cost of additional water. Customer demand further constrained frequent hygiene practices, as delays could lead to loss of sales. These findings demonstrate that infrastructure limitations and economic pressures directly impact hygiene compliance.

Regular inspections by health professionals were strongly associated with good practices, consistent with findings from Gedeo Zone, Southern Ethiopia, and Kiambu County, Kenya [18, 28, 38]. Qualitative findings indicate that oversight reinforces hygiene standards, increases accountability, and encourages vendors to maintain safe practices even when economic or customer pressures conflict with hygiene behaviors. In the absence of supervision, vendors often underestimated the importance of gloves, aprons, or proper food handling, highlighting the role of structured guidance in shaping behavior.

Vendors with good food hygiene knowledge were more likely to practice safe handling, consistent with findings from Shashamene, Gondar city, and Myanmar [17, 20, 24]. Qualitative data suggest that many vendors lacked awareness of basic food safety principles, including separating raw and cooked foods, frequent hand washing, and proper storage conditions. Misconceptions, such as assuming food is safe if it looks or smells acceptable, contributed to unsafe practices. These insights explain the observed quantitative association and highlight the potential impact of targeted educational interventions.

Formal food handling training was associated with better hygiene practices, in line with studies from Dessie, Can Tho city, Nigeria and Ghana [17, 19, 22, 38, 39]. Qualitative findings indicate that vendors relying on informal learning often adopted unsafe habits due to lack of structured guidance and limited access to institutional support. Those receiving formal training demonstrated clearer understanding of cross-contamination, utensil hygiene, and storage practices and applied these consistently. These findings emphasize that structured training enhances both knowledge and practical skills, supporting safer hygiene behaviors.

This study has some limitations that should be considered when interpreting the findings. First, hygiene practices may have been influenced by behavior modification due to direct observation, and self-reported measures are subject to social desirability bias. Second, the cross-sectional study design limits the ability to infer causality. Although the analysis identified significant associations between hygiene practices and factors such as food safety training, work experience, access to continuous water supply, regular health inspections, and food hygiene knowledge, these relationships should not be interpreted as causal. The observed associations may be influenced by unmeasured confounding or reverse causation.

This study revealed that a relatively low proportion of street food vendors in Addis Ababa practiced adequate food hygiene. Quantitative findings identified key factors associated with better hygiene practices, including formal food safety training, adequate food hygiene knowledge, uninterrupted water supply, regular professional supervision, and more than 1 year of work experience. Qualitative insights further highlighted structural and operational barriers, such as limited water and sanitation infrastructure, poor environmental conditions, financial constraints, and customer pressure, which collectively hinder consistent hygiene compliance.

These findings provide a strong basis for targeted interventions to improve food hygiene among street food vendors. Structured, practice-oriented food safety training programs could enhance vendors’ knowledge and practical skills, particularly among newer or less experienced vendors. In parallel, strengthening municipal inspection and supportive supervision systems may reinforce hygiene standards through regular monitoring and feedback. Addressing infrastructural and economic constraints such as improving access to reliable water, sanitation facilities, and affordable hygiene supplies would further enable vendors to adopt and sustain safe food handling practices.

Future research should employ longitudinal designs to assess causal relationships between identified factors and hygiene practices over time. In addition, intervention studies evaluating the effectiveness of training programs, regulatory enforcement, and infrastructure improvements are recommended to generate evidence on scalable strategies for improving street food hygiene in urban settings.
